# The Role of Ph Fronts in Tissue Electroporation Based Treatments

**DOI:** 10.1371/journal.pone.0080167

**Published:** 2013-11-21

**Authors:** Felipe Maglietti, Sebastian Michinski, Nahuel Olaiz, Marcelo Castro, Cecilia Suárez, Guillermo Marshall

**Affiliations:** 1 Laboratorio de Sistemas Complejos, Departamento de Computación, Facultad de Ciencias Exactas y Naturales, Universidad de Buenos Aires/Consejo Nacional de Investigaciones Científicas y Técnicas (CONICET), Buenos Aires, Argentina; 2 Instituto Tecnológico de Buenos Aires (ITBA), Buenos Aires, Argentina; 3 Grupo de Investigación y Desarrollo en Bioingeniería, CONICET/Universidad Tecnológica Nacional (UTN/Facultad Regional Buenos Aires (FRBA), Buenos Aires, Argentina; National Research Council, Italy

## Abstract

Treatments based on electroporation (EP) induce the formation of pores in cell membranes due to the application of pulsed electric fields. We present experimental evidence of the existence of pH fronts emerging from both electrodes during treatments based on tissue EP, for conditions found in many studies, and that these fronts are immediate and substantial. pH fronts are indirectly measured through the evanescence time (ET), defined as the time required for the tissue buffer to neutralize them. The ET was measured through a pH indicator imaged at a series of time intervals using a four-cluster hard fuzzy-c-means algorithm to segment pixels corresponding to the pH indicator at every frame. The ET was calculated as the time during which the number of pixels was 10% of those in the initial frame. While in EP-based treatments such as reversible (ECT) and irreversible electroporation (IRE) the ET is very short (though enough to cause minor injuries) due to electric pulse characteristics and biological buffers present in the tissue, in gene electrotransfer (GET), ET is much longer, enough to denaturate plasmids and produce cell damage. When any of the electric pulse parameters is doubled or tripled the ET grows and, remarkably, when any of the pulse parameters in GET is halved, the ET drops significantly. Reducing pH fronts has relevant implications for GET treatment efficiency, due to a substantial reduction of plasmid damage and cell loss.

## Introduction

Cancer is one of the leading causes of death worldwide, accounting for 7.6 million deaths in 2008 and it is projected to continue rising, with an estimate of 13.1 million deaths in 2030 [1]–[2]. Cancer treatment requires a careful selection of one or more interventions (such as surgery, radiotherapy and chemotherapy). The goal is to cure the disease or considerably prolong life while improving the patient's life quality. To improve efficiency and reduce side effects, during the last decades a considerable number of new therapies have been presented, many of which are currently under study. Among them, new tumor treatments based on electroporation (EP) emerged during the last years, becoming a new step in cancer treatment.

EP induces pore formation across the cell membrane by the application of an electric field [3]–[4]. During the last decades EP-based techniques were implemented for medical purposes [5]–[6]. Theoretical and experimental studies about EP-based techniques were conducted in the 1970s [7]–[9] and Neumann et al. [10] reported for the first time a successful gene transfer into murine cells using EP. After the development of EP devices this technique became widespread for delivering molecules inside the cell [11]–[13]. The first use of EP for delivering chemotherapeutic agents in tumors was reported by Belehradek in 1993 [14]. Since then many tumor treatment modalities using EP, delivering genes or drugs, have been developed [15]–[19].

Electrochemotherapy (ECT) is an EP-based technique in which electric pulses are used to induce transient pore formation across the cell membrane, thus allowing certain drugs, which are non permeant to it, to enter the cell [3]–[4], [20]–[21]. ECT is being used as a therapeutic option for cutaneous and subcutaneous tumors since 2006, after its clinic standard operating procedures were published [22]–[23], and recently a meta-analysis has been published [24]. A typical ECT treatment in humans consists of a train of 8 square pulses of a high electric field (around 1000 V/cm) and short duration (around 100 µs) delivered at 1 Hz [3]. Gene Electrotransfer (GET, formerly EGT) is another EP-based technique that uses the same principle to transfer plasmidic DNA instead of a drug [25]–[26]. For sufficiently strong electric fields, pores can remain permanently open, thus killing the cell. This is the case of irreversible electroporation (IRE) when the cell membrane does not reseal [27]. Another type of tissue electroporation is nanoelectroporation, which is characterized by the use of pulses of nanosecond length. In this case the pores are formed in the organelles, producing a massive calcium release to the cytoplasm which triggers cell death mechanisms [28]–[29].

EP-based treatments aim mainly at a palliative care, providing a new treatment modality where others have failed. ECT can be used also as a neoadjuvant treatment or to extend surgical margins [30]–[31]. Additionally efforts are being made to increase the local immune response generated by the treatment in order to obtain a systemic response [26], [32]. Many efforts are geared towards the extension of EP-based treatments to other organs such as the brain [33], [59], the liver [34], the lungs [35] and the bones [36]. Moreover, an endoscopic electrode was developed for endoluminal applications [37]. GET has become an efficient and safe transfection method in medicine to treat many diseases. Among them it can be used to produce a protein which is deficient due to a genetic abnormality, or a protein with a therapeutic effect. Clinical trials have been performed in Parkinson's disease, retinoblastoma, age related macular degeneration, cystic fibrosis, coronary artery disease, peripheral vascular disease, muscular dystrophies, junctional epidermolysis bullosa, HIV/AIDS, hemophilia, severe combined immunodeficiency, α_1_-antitrypsin deficiency and cancer [30], [26]. Recently a phase I clinical trial in patient with metastatic melanoma concluded with very promising results [32].

Whether ECT, GET or IRE, all these techniques have undesired side effects (loss of cell viability, uncontrolled necrosis, plasmid damage) that must be minimized. To elucidate how much electric pulses can change the pH solution in the electroporation of a cell suspension, the changes of pH in NaCl solutions buffered with different amount of sodium phosphate under high voltage electric pulses was studied in [38]. Using stainless steel anode and different materials in the cathode, it was found that variations in the whole volume of the electrolytic chamber can exceed 1–2 pH units in average, though it was observed that near the cathode this variation is significantly greater. It was also conjectured that the change of pH, in some cases, might be one of the factors causing cell death. In conclusion, to minimize pH effects, it was recommended to use strongly buffered solutions and bipolar electric pulses. In a previous paper [39] we looked into the electroporation process from a new angle apparently overseen in the literature, the role of pH in ECT modeling based on ion transport during the treatment. The analysis was developed through in vitro gel measurements and theoretical modeling drawing from previous experience in the electrochemical treatment of tumors (EChT, another electrochemical-based antitumoral treatment that uses constant electric fields with the aim of eliminating tumors mainly by necrosis, see for instance [39]–[43], [58]) and in electrochemical deposition in thin layer cells [44]. It is well known that, during EChT, two opposing pH fronts emerge from both electrodes (acid from the anode and basic from the cathode) until there is a collision somewhere between them. These pH fronts can be used to predict the extent of the tumor necrotic area [45] which may be, in part, attributed to electrodenaturation [43]. While in EChT tumor necrosis is the main goal of the treatment, and in IRE it contributes to tumor destruction, in ECT and GET, it is an undesired effect. On the other hand, although GET has high in-vitro efficiency, it is far from its optimal efficiency in vivo, compared to other transfection methods [32]–[46]. It has been suggested that both effects (uncontrolled necrosis and loss of efficiency) may be strongly dependent on pH alterations induced by electrolysis during the process. Significant pH changes of the medium may induce deleterious effects over the plasmids used in GET, as DNA denaturation is prominently affected by pH. Plasmids are usually denatured when exposed to an alkaline medium above pH 8.4, and it takes less than 1 second to permanently denaturate them at pH 12.5 [47]–[49]. Thus, it seems relevant to quantify the extent and evolution of pH changes during EP-based treatments. A way to reduce the effects of pH changes could be by minimizing voltages and the number of pulses while maximizing pulse length as far as possible. In fact, this new low-current, low-voltage and long-duration pulse procedure was proved recently to be safer and more efficient in DNA and GET [5], [50], [26].

As an extension of our previous results [39], the aim of this paper is to show that pH fronts generated by EP-based techniques produce non negligible pH changes in a tissue regardless of the presence of natural buffers. pH fronts are measured through the evanescence time (ET), that is, the time required for the tissue buffer to neutralize them. The ET is studied for different sets of pulse parameters (corresponding to IRE, ECT or GET modalities).

## Materials and Methods

**Figure 1:**
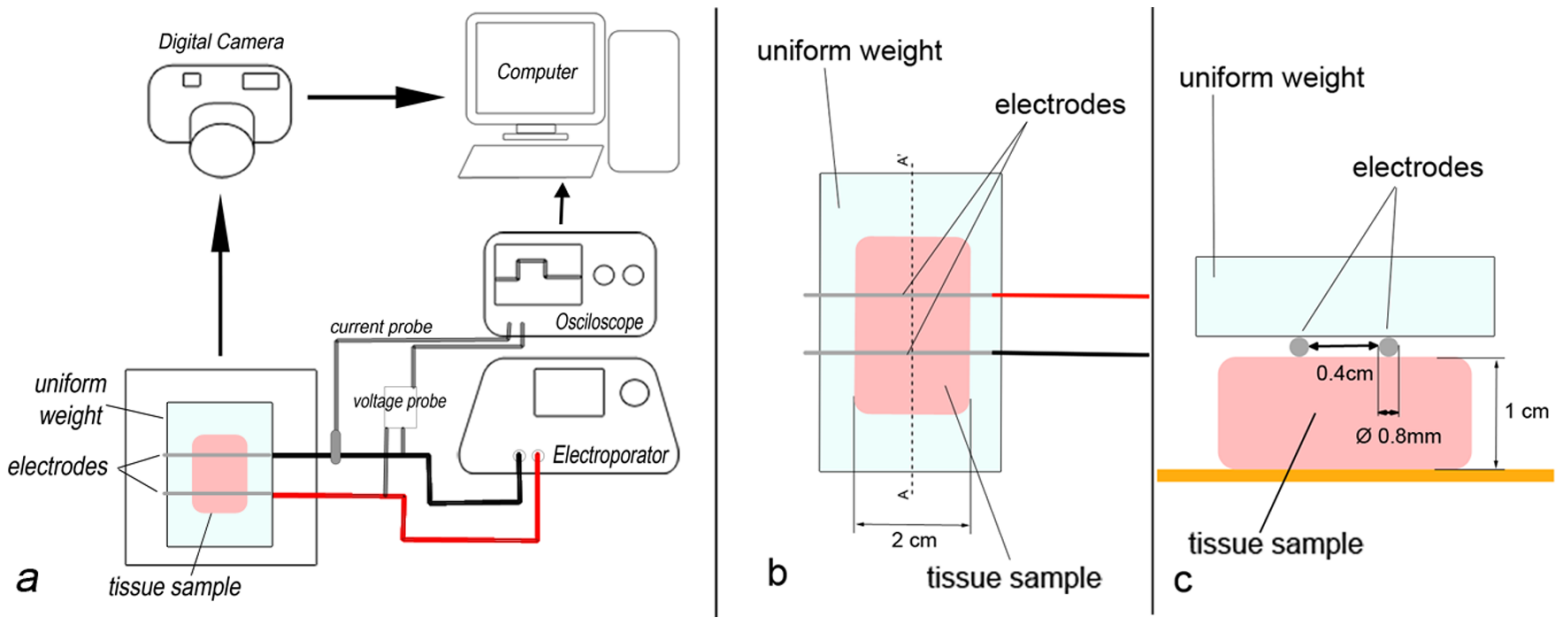
Experimental setup. a. the two-needle electrode was placed over the tissue sample (under a uniform weight) and connected to the electroporator, during the pulses the electric current and potential were monitored with an oscilloscope, color changes in the dye after the pulses were recorded with a video camera and analyzed in a PC; b. zoom of the tissue sample with the two-needle electrode placed over it; c. cross section (A-A′) of the tissue sample and the two-needle electrode.

Experimental measurements are based on the application of EP under different pulse parameters to chicken muscular tissue. Induced pH effects are measured indirectly by the evanescence time of a pH indicator. The experimental setup is shown in figure 1. Muscular tissue samples of 3×2×1 cm were sliced from fresh chicken (no ethical approval was required since the ex vivo experiments where performed with chicken muscular tissue acquired from a retailer). All cuts were made right before each data acquisition in order to prevent the tissue from drying out. To estimate the hydroxyl production around the cathode inside the tissue, a pH indicator dye (phenolphthalein, C_20_H_14_O_4_, transition pH range 8.0–9.6, from colorless to red) was used, each sample placed in a plastic plate. One drop of phenolphthalein was applied over the surface of the samples and two electrodes (parallel surgical steel needles 0.8 mm thick, 2.5 cm long, their surfaces separated from each other by 0.4 cm) were laid horizontally over the region of interest. The surgical steel needles were chosen since it is the material used in many publications and in clinical practice, also because it induces less pH changes [51]–[52]. In all of the experiments, a uniform weight of 46.6 grams was applied on top of the needles to exert the same pressure over the tissue. Only the hydroxyl production at the cathode was studied as it is equivalent to the proton production at the anode by the following hydrolysis stechiometric equations [40], [52]:

At the anode,
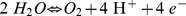



At the cathode,
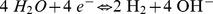



The oxidation of the anion of the solute,
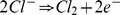



The electric pulses were applied using a BTX ECM 830 square wave electroporator (Harvard Apparatus, Massachusetts, USA). Immediately after pulse delivery, electrodes were removed allowing video capturing of the image area by a CASIO EX-FH25 high-speed camera. Videos were recorded at 30 fps with a 1280×720 pixel resolution. For ET estimation, one frame per second was considered and, in each frame, the region of interest was manually cropped around the cathode. The dye is better depicted in the green channel and it was therefore chosen as the only component for analysis (ImageJ, http://rsbweb.nih.gov/ij). This choice produced a more accurate segmentation. Immediately after the onset of pulse delivery, the pH indicator dye in the sample changes from colorless to red. After the end of the pulse delivery and as hydroxyls are neutralized by the tissue buffer, the sample turns to colorless again, indicating a neutralizing phenomenon. We define ET as the time when 90% of the dye changed from red to colorless after the end of the pulse delivery. This ET, which is an indirect measure of the amount of hydroxyls produced, is estimated by the time needed for the tissue buffer to turn the pH value to less than 8 (nearly neutral). To make this measure accurate and reproducible, real time videos of the samples after pulse delivery were captured showing the color change of the dye over time. Afterwards, the video was analyzed to automatically determine the time required for 90% of the dye to turn colorless.

Different sets of pulse parameters, corresponding to different experimental series, were studied by changing the standard number of pulses, pulse length and pulse amplitude. During all the experiments electric current and potential were measured using an Agilent DSOX2012A Oscilloscope: 100 MHz, 2 Channels (Agilent Technologies, Santa Clara, CA, USA). A statistical analysis of the results was made by ANOVA and a p<0.01 was considered significant.

**Figure 2:**
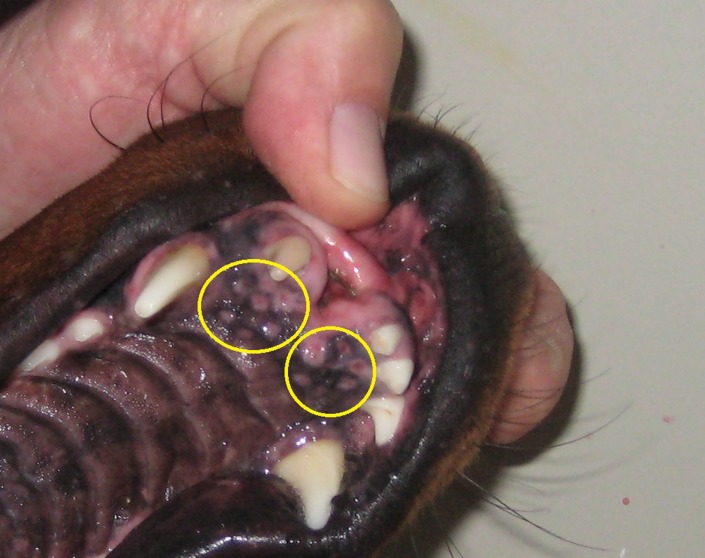
Minor tissue injuries observed seven days after a standard ECT treatment in the mouth of a canine patient. Injuries are evidenced as light red halos surrounding central red spots in the mucosa (encircled in yellow). The dog owner's hand is shown in the picture.

The treated animal shown in figure 2 is not from a laboratory or from research; it is a veterinary patient with a spontaneous disease, whose only alternative was euthanasia. This is why the veterinary doctor decided to perform ECT. Because it was a veterinary patient and the decision of making a treatment was not based on research but on disease treatment, the owner's consent was obtained. Also, owner's consent was obtained to use the dog's image in scientific work. In any case, all recommendations by the Consejo Profesional de Médicos Veterinarios de Buenos Aires were followed. The local normative law in Argentina, law No. 14072 regulating the practice in veterinary medicine, was followed too.

## Results and Discussion

As previously discussed, experimental measurements are based on the application of EP under different pulse parameters to chicken muscular tissue.

The parameters for GET found in the literature are very diverse [26]. Here we followed those suggested in [25] because we were familiar with them, and also because we applied them in other experiments with good results. Parameter variations were chosen according to the expected variations in the pH changes, in order to confirm that a lower Coulomb dose calculated will correspond with a lesser pH change in the tissue, and a higher Coulomb dose will induce the opposite. The frequency was kept as high as possible in order to diminish patient discomfort in future applications. Higher frequencies are preferred in clinical practice since they induce lesser muscle contractions and reduce treatment time, diminishing patient discomfort [33], [53]–[54]. Induced pH effects are measured indirectly by the evanescence time of a pH indicator. Table 1 presents all pulse parameters tested and the corresponding Coulomb dose. A surrogate of the Coulomb dose (here on Coulomb dose) is calculated as electric current density times pulse duration times number of pulses delivered. Each experimental series was repeated four times. The standard set of pulse parameters for ECT, GET and IRE were obtained from the literature (experimental series 1, 2 and 3) [22], [25], [33]. The GET test consists of one high voltage (HV) pulse followed by four low voltage (LV) pulses. Since the HV pulse did not produce significant changes in the tissue (from previous experiments data, not shown here), only the four LV pulses were taken into account. Electric pulses were delivered at the maximum frequency allowed by the thermal damage threshold. Nevertheless, to avoid tissue thermal damage during an IRE application, the frequency was kept at 1 Hz. The rise of temperature in the tissue due to pulse delivery was not studied, although we speculate that it is not negligible. In fact, it will probably contribute to more plasmid denaturation and tissue damage, and be more significant in the case of higher pulse parameters [55]–[57].

**Table 1:** Pulse parameters for each experimental series and the average evanescence time obtained (ET).

Experimental series	Voltage-to-distance ratio [V/cm]	Pulse length [ms]	Number. Of pulses	Interval [ms]	Frequency [Hz]	Surrogate Coulomb dose [C/cm^2^]	Evanescence time [s]	Description
1	1500	0.1	90	1000	1	0.0317	<5	IRE Standard
2	1000	0.1	8	100	10	0.0019	<5	ECT Standard
3	80	100	4	100	5	0.1	27	GET Standard
4	160	100	4	100	5	0.2	62.8	GET Double pulse amplitude
5	80	200	4	100	3.33	0.2	56.8	GET Double pulse length
6	80	100	8	100	5	0.2	47.25	GET Double number of pulses
7	240	100	4	100	5	0.304	70.83	GET Triple pulse amplitude
8	80	300	4	100	2.5	0.304	71	GET Triple pulse length
9	80	100	12	100	5	0.304	114.4	GET Triple number of pulses
10	40	100	4	100	5	0.0416	11.67	GET Half pulse amplitude
11	80	50	4	100	10	0.0416	17	GET Half pulse length
12	80	100	2	100	10	0.0416	21	GET Half number of pulses

**Figure 3:**
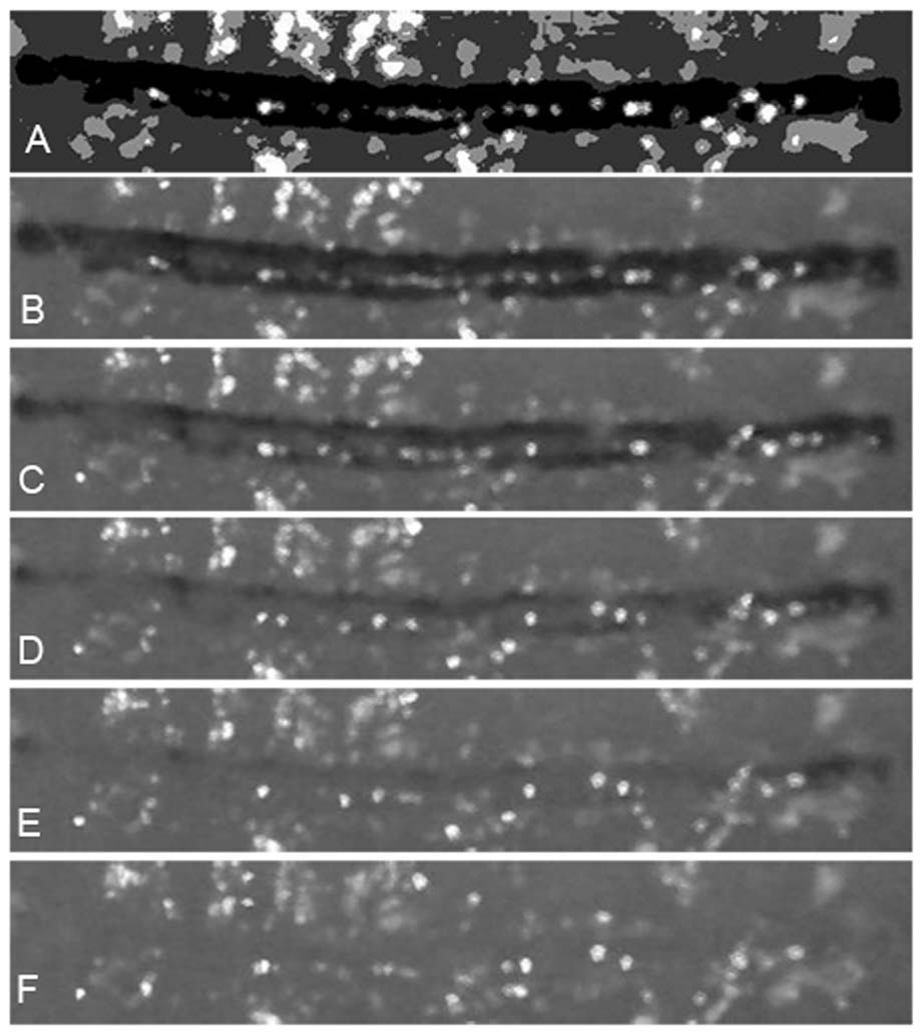
Typical set of images obtained and the way they were processed. The initial frame immediately after pulse delivery (frame 3B) was used to generate a segmented image (frame 3A). Frames 3B to 3F show the dye turning colorless. Frame 3F shows the stage at which the pH change was completely neutralized by tissue buffer.

Figure 3 illustrates a typical set of images obtained and the way they were processed. The initial frame immediately after pulse delivery (figure 3 B) was used to generate a segmented image (figure 3 A). In this image the region where the pH change took place was automatically identified and set to the lowest intensity value (dark region) by means of a four-class hard fuzzy-c-means algorithm (MIPAV, http://mipav.cit.nih.gov). For each experiment, the intensity-based clustering algorithm finds the optimum threshold separating different classes of intensity ranges (4 categories in figure 3 A) and the dye matching one of these categories is chosen. In order to monitor the pH neutralization process the number of pixels whose intensity remained within that range was calculated at each video frame using an Octave script (GNU Octave 3.6.2). Figure 3 B–F shows snapshots of the dye turning colorless. The last image, figure 3 F corresponds to the stage at which the pH change is completely neutralized by the tissue buffer.

**Figure 4:**
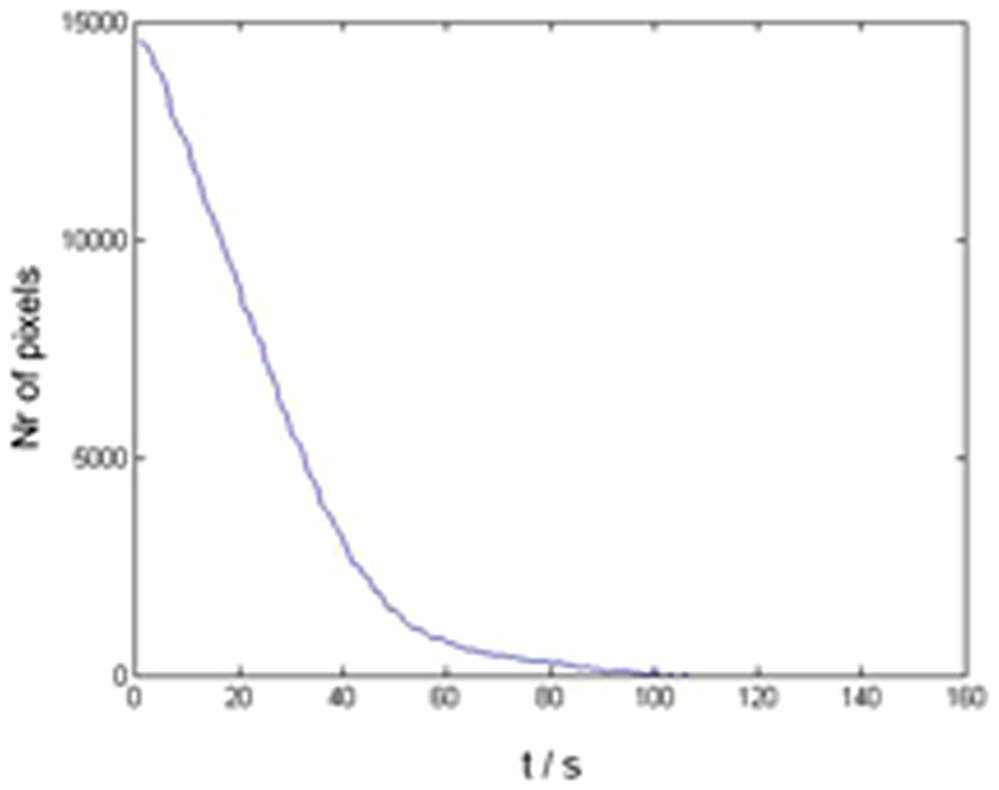
Time evolution of the number of pixels corresponding to the dye after a GET of 4 pulses of 160/cm, 100 msec long, at 5 Hz was applied.

Figure 4 shows the evolution of the number of pixels corresponding to the dye in time, in this case for a GET application with 4 pulses of 160 V/cm, 100 msec long, at 5 Hz. As seen in the figure, as the dye turns colorless the algorithm counts an increasingly smaller number of pixels that meet the criterion. Finally, when the dye completely turned colorless, no pixel of intensity within the range was present. The ET was defined as the time when the number of pixels corresponding to the dye was reduced by 90%.

**Figure 5:**
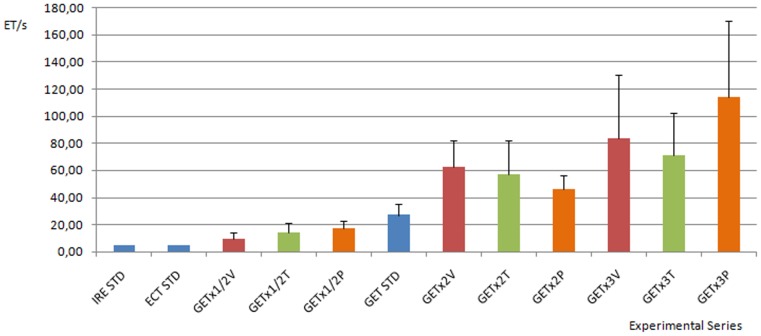
Chart of the mean evanescence time ET (s) for each experimental series. The standard pulse parameters for IRE, ECT and GET are depicted in blue. Pulse parameters corresponding to half, double and triple standard GET pulse amplitudes are shown in red. The pulse parameters corresponding to half, double and triple standard GET pulse lengths are depicted in green. Finally, pulse parameters corresponding to half, double and triple standard GET number of pulses are shown in orange. Standard IRE, standard ECT and all GET parameter variations are significantly different from standard GET (p<0.01, N = 6). All experimental series are markedly different from each other (p<0.01, N = 6), the bar represents mean evanescence time, error bars show standard deviation.

Figure 5 presents a chart of ET for each experimental series. Optimal pulse parameters for IRE, ECT and GET are depicted, as well as the different experimental series (described in detail in table 1). It is observed that pH changes in GET last longer than in ECT and IRE, ranging from 30 seconds to about 2 minutes. Even when standard GET pulse parameters are considered, plasmid exposition times at these extreme pH conditions are enough to produce their denaturation. When any of the pulse parameters (pulse length, pulse amplitude or number of pulses) is halved, ET is significantly lowered as compared with standard GET. This result may have relevant implications for treatment efficiency, since less exposition time results in less plasmid damage and less cell loss. Also, there is a significant increase in the ET when doubling or tripling pulse amplitude, pulse length or the number of pulses, as compared with standard GET. It is important to note that the electric field, current density and heating distributions are nonlinear (spatially distributed), thus electrochemical reactions and pH changes. Nevertheless in this work we focused in the strongest pH changes produced in the vicinity of the electrodes. Higher Coulomb doses correlate with stronger pH changes and thus with longer evanescence times. This could contribute to less tissue sparing in ECT and less plasmid expression in GET. These results suggest that the effect of pulse parameters variation determining the Coulomb dose and thus pH fronts must be taken into account to improve GET efficiency, make more accurate treatment planning and design models that predict the outcome of different GET protocols better.

**Figure 6:**
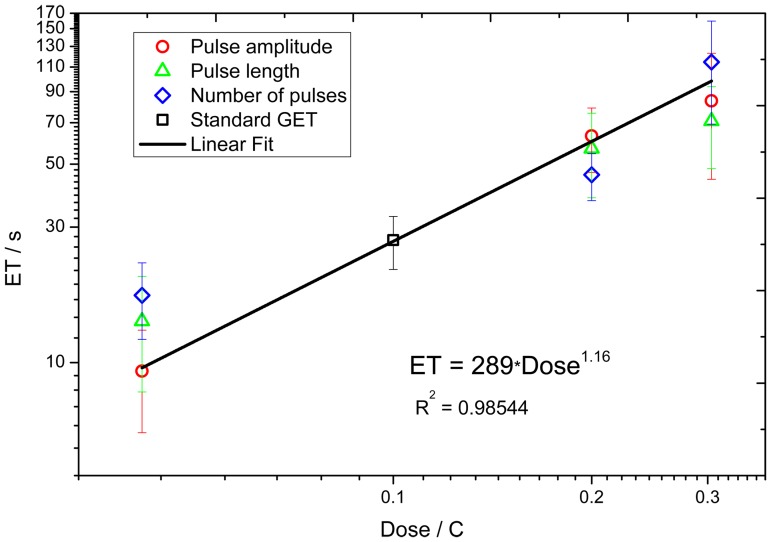
Graph of ET vs. applied surrogate Coulomb dose, for GET protocols. The squares represent the ET for standard GET parameters, the circles represent mean ET for half, double and triple pulse amplitudes respectively. The triangles represent mean ET for half, double and triple pulse lengths respectively. The rhombuses represent mean ET for half, double and triple number of pulses. Error bars show standard error.

Figure 6 presents a log-log scale of ET as a function of the applied Coulomb dose corresponding to GET protocols. A linear regression analysis shows a linear law of ET as a function of Coulomb dose, i.e. ET = 289 D^1.16^ with an R-squared of 0.985. The graph shows that ET scales almost linearly with the Coulomb dose. This may be relevant in clinical applications, since knowing the Coulomb dose allows estimation of the ET value and thus, a priori knowledge of whether pH effects are admissible.

ET depends not only on the Coulomb dose applied but also on the way it is applied. For instance doubling the voltage would be the same as doubling the pulse length in terms of the Coulomb dose, but not in terms of evanescence time where doubling the voltage produces longer evanescence times. This result in tissues has been previously reported in gels [38]. One possible reason of this discrepancy is that by applying the same current divided in more pulses, the sum of the time between pulses is larger, so there is a longer time for the diffusion of electrolysis end products outside the electrode zone (gas bubbles isolating the needles and the other electrolytes such as hydrogen and hydroxyls). Also, there is a longer time for water molecules to reach the area between the electrodes and be the substrate for the electrolysis in the next pulse delivery. Further experiments are needed to corroborate these conjectures.

Table 1 and figure 5 show that the ET induced by standard ECT and IRE lasts a very short time, usually less than 5 seconds, though enough to contribute to tissue necrosis near the electrodes. This might be irrelevant in IRE (all tissue near electrodes is intended to be ablated), but not in ECT where the treatment must be as specific to tumor cells as possible. As an example, figure 2 shows minor tissue injuries observed seven days after a standard ECT procedure (8 pulses, 1000 V/cm, 100 us, 1 Hz) was applied to a canine patient (an 8 year-old male Doberman) with a squamous cell carcinoma located in the anterior part of the mouth. The area depicted in the figure corresponds to healthy tissue margins treated to prevent tumor recurrence (security margins). Tissue injuries are evidenced as light red halos surrounding central red spots in the mucosa (corresponding to points where electrodes were placed). These injury halos can be the consequence, at least partially, of the extreme pH changes induced by the electric pulses applied.

## Conclusions

EP-based treatments have proven its efficacy in clinical medicine, and became a valuable option where other treatments failed or were not applicable. GET is an effective technique for delivering genes inside the cell, and very promising for treating not only cancer related diseases but also a wide spectrum of other pathologies. Though lacking the side effects and potential dangers of viral vector transfection techniques, it's in vivo efficiency is still rather poor compared to them. Nevertheless, there is still room for improvement. Although many efforts have been made in recent years to fully understand the basic mechanism of GET, there are very few publications studying the effects of pH changes in GET, a phenomenon which is very well studied in other problems involving electric fields. Here we presented experimental evidence of the existence of substantial pH fronts during GET procedure in conditions that are typical in many studies found in the literature. pH fronts were measured through the ET, that is, the time required for the tissue buffer to neutralize them. While in EP-based treatments such as ECT and IRE, the ET is very short, in GET it is much longer. This raises the risk of plasmid denaturalization and cell damage with the consequent loss of GET efficiency. We speculate that reducing pH fronts effects, as far as possible, may have relevant implications for GET efficiency. These tissue pH effects must be taken into account for more accurate treatment planning and model design, and to predict the outcome of the application for predefined pulse parameters in different tissues better. Understanding and controlling pH in EP-based treatments may significantly contribute an increase in its effectiveness. This will also help to achieve a better understanding of the whole electropermeabilization phenomenon.
